# Cranial irradiation induces transient microglia accumulation, followed by long-lasting inflammation and loss of microglia

**DOI:** 10.18632/oncotarget.12929

**Published:** 2016-10-26

**Authors:** Wei Han, Takashi Umekawa, Kai Zhou, Xing-Mei Zhang, Makiko Ohshima, Cecilia A. Dominguez, Robert A. Harris, Changlian Zhu, Klas Blomgren

**Affiliations:** ^1^ Department of Pediatrics, Henan Provincial Women's and Children's Hospital, Zhengzhou, P.R. China; ^2^ Henan Key Laboratory of Child Brain Injury, The Third Affiliated Hospital of Zhengzhou University, Zhengzhou, P.R. China; ^3^ Karolinska Institutet, Department of Women's and Children's Health, Stockholm, Sweden; ^4^ Department of Obstetrics and Gynecology, Mie University Graduate School of Medicine, Tsu, Japan; ^5^ Karolinska Institutet, Department of Clinical Neuroscience, Center for Molecular Medicine, Karolinska University Hospital, Stockholm, Sweden; ^6^ Center for Brain Repair and Rehabilitation, Institute of Neuroscience and Physiology, University of Gothenburg, Gothenburg, Sweden; ^7^ Department of Pediatric Oncology, Karolinska University Hospital, Stockholm, Sweden

**Keywords:** irradiation, neurogenesis, neuroinflammation, microglia, monocyte, macrophage

## Abstract

The relative contribution of resident microglia and peripheral monocyte-derived macrophages in neuroinflammation after cranial irradiation is not known. A single dose of 8 Gy was administered to postnatal day 10 (juvenile) or 90 (adult) CX3CR1^GFP/+^ CCR2^RFP/+^ mouse brains. Microglia accumulated in the subgranular zone of the hippocampal granule cell layer, where progenitor cell death was prominent. The peak was earlier (6 h vs. 24 h) but less pronounced in adult brains. The increase in juvenile, but not adult, brains was partly attributed to proliferation. Microglia numbers then decreased over time to 39% (juvenile) and 58% (adult) of controls 30 days after irradiation, largely as a result of cell death. CD68 was expressed in 90% of amoeboid microglia in juvenile hippocampi but only in 9% of adult ones. Isolated hippocampal microglia revealed reduced CD206 and increased IL1-beta expression after irradiation, more pronounced in juvenile brains. CCL2 and IL-1 beta increased after irradiation, more in juvenile hippocampi, and remained elevated at all time points. In summary, microglia activation after irradiation was more pronounced, protracted and pro-inflammatory by nature in juvenile than in adult hippocampi. Common to both ages was long-lasting inflammation and the absence of monocyte-derived macrophages.

## BACKGROUND

As a commonly used treatment modality for brain tumors, cranial irradiation (IR) also causes debilitating cognitive decline, particularly in children, but also in adults, especially when radiation fields involve the temporal lobe, where the hippocampus is located [1–4]. The mechanisms underlying the cognitive deficits are only partly known, but likely include reduced hippocampal neurogenesis [5, 6]. It has been shown in rodents that hippocampal long-term memory formation depends on neurogenesis [7, 8]. We and others have shown that IR-induced reduction of neurogenesis is persistent and progressive [9–11] and amelioration of cognitive impairment could be achieved by restoring neurogenesis through physical exercise [12] or lithium treatment [13].

The primary purpose of an acute inflammatory response is to defend against noxious stimuli and restore neural integrity in the injured brain. However, uncontrolled and sustained inflammation exacerbates the neuronal injury [14]. It has been shown in rodents that IR-induced chronic inflammation contributes to the persistent, and even progressive [15] impairment in neurogenesis [16, 17]. It has been suggested that cranial irradiation causes chronic inflammation and that this contributes to the accelerated neurodegeneration and cognitive decline observed in patients [18]. In pediatric oncology, this is particularly problematic given the increased vulnerability of the growing brain and the long life expectancy of the patients who are cured from their disease. Very little is known about the role of neuroinflammation in the increased susceptibility of the juvenile brain to ionizing radiation, and we were particularly interested in the possible contribution of immune cells recruited from the blood stream.

At least two mechanisms causing reduced neurogenesis after radiotherapy can be distinguished: direct depletion of neuronal progenitors through cell death caused by DNA double strand breaks, mainly apoptosis [19], and IR-induced neuroinflammation altering the microenvironment, causing a shift from neurogenesis to gliogenesis [17]. Accordingly, IR-induced neurogenesis impairment was exacerbated in animals with systemic inflammation [20], and anti-inflammatory treatment could partially restore neurogenesis and mitigate IR-induced cognitive deficits [16, 21]. Neuroinflammation represents the inflammatory response originating in the CNS as a consequence of brain injury, characterized by the activation of glial cells with the consequent production of pro-inflammatory agents, and may also involve infiltrating blood-borne immune cells. The neuroinflammation induced by IR, as judged by activation of microglia, varies depending on the brain developmental stage [17, 22, 23]. It has been shown that a single IR dose of 10 Gy can cause a persistent increase in microglia proliferation and activation in the adult rat [17]. In the juvenile rat brain, however, the response has been demonstrated to be transient, with microglia numbers increased as soon as 6 hours after the insult and decreased to lower than normal levels after 1 week [24]. Expression of the pro-inflammatory chemokine CCL2 (also known as monocyte chemoattractant protein- 1 (MCP-1), was transiently up-regulated in both young and adult rat brains [23, 25].

Macrophages, as the first line of defense, play a critical role under pathological conditions through antigen presentation and their production of cytokines and growth factors [26–28]. In the injured brain, the macrophage population may consist of both resident microglia and infiltrating monocyte-derived macrophages [29–31]. Monocytes expressing the chemokine receptor CCR2 can be selectively recruited into injured tissue through the regulation of CCL2, and become macrophages [31, 32]. The finding that CCL2 deficiency was sufficient to restore hippocampal neurogenesis after cranial irradiation suggested that pro-inflammatory monocytes might contribute to the persistent neuroinflammation after IR [25]. However, given the lack of specific markers, it is difficult to distinguish resident microglia from macrophages derived from blood-borne, peripheral monocytes using conventional techniques. Little is known about the relative contributions of resident microglia and infiltrating macrophages in the inflammatory response at different ages. A better understanding of these two evolutionarily different groups of macrophages and antigen-presenting cells is important in the search for new targets for clinical interventions.

**Figure 1:**
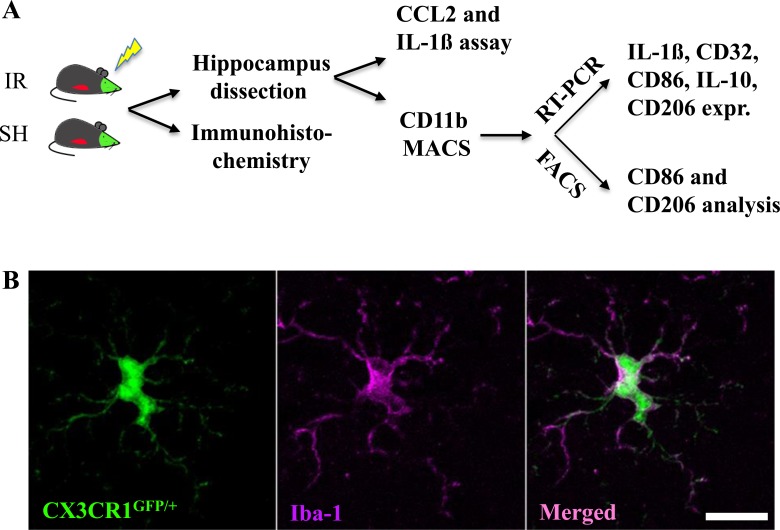
Experimental design Reporter mice, CX3CR1^GFP/+^ and CCR2^RFP/+^, expressing GFP in resident microglia and RFP in monocyte-derived macrophages, were subjected to irradiation (IR) or sham procedures (SH). Hippocampal tissue was subjected to immunohistochemistry and stereological quantification, or analyzed for CCL2 and IL-1ß protein using ELISA. Microglia were isolated from the hippocampus using MACS and further analyzed using either RT-PCR or FACS (**A).** All CX3CR1^GFP/+-^labeled cells expressed Iba-1 in sham control brains, and all Iba-1-positive cells were positive for CX3CR1^GFP/+^. Scale bar, 10 μm (**B).**

The discovery of the fractalkine receptor CX3CR1 has facilitated the exploration of microglial and monocyte function [33]. In the CNS, CX3CR1 is expressed exclusively in microglia [15]. In the periphery, it is also present on “resident” monocytes, dendritic cells, NK cells and T cells [15], but not on “inflammatory” monocytes phenotypically identified as Ly6C^hi^CCR2^+^CX3CR1^−^ commonly found in inflamed tissues [32, 34]. In the current study, we used a reporter mouse (CX3CR1^GFP/+^CCR2^RFP/+^), where microglia (CX3CR1^+^) are labeled with green fluorescent protein (GFP) and monocyte-derived macrophages (Ly6C^hi^CCR2^+^CX3CR1^−^) are labeled with red fluorescent protein (RFP) [35] to characterize the responses of these two evolutionarily different groups of macrophages after IR in both juvenile and adult brains. The experimental design is outlined in Figure 1A.

## RESULTS

### No infiltrating monocytes, neither in the juvenile nor in the adult hippocampus after IR

CX3CR1^GFP/+^ was exclusively present in microglia and was not detectable in any other type of cells, neither in sham nor in irradiated brains, as shown by consistent co-localization with the microglial marker Iba-1 (Figure 1B). Conversely, all Iba-1-positive cells were also positive for CX3CR1^GFP/+^. No CCR2^RFP/+^ expression was detected in the parenchyma of sham-irradiated brains in any brain region.

**Figure 2:**
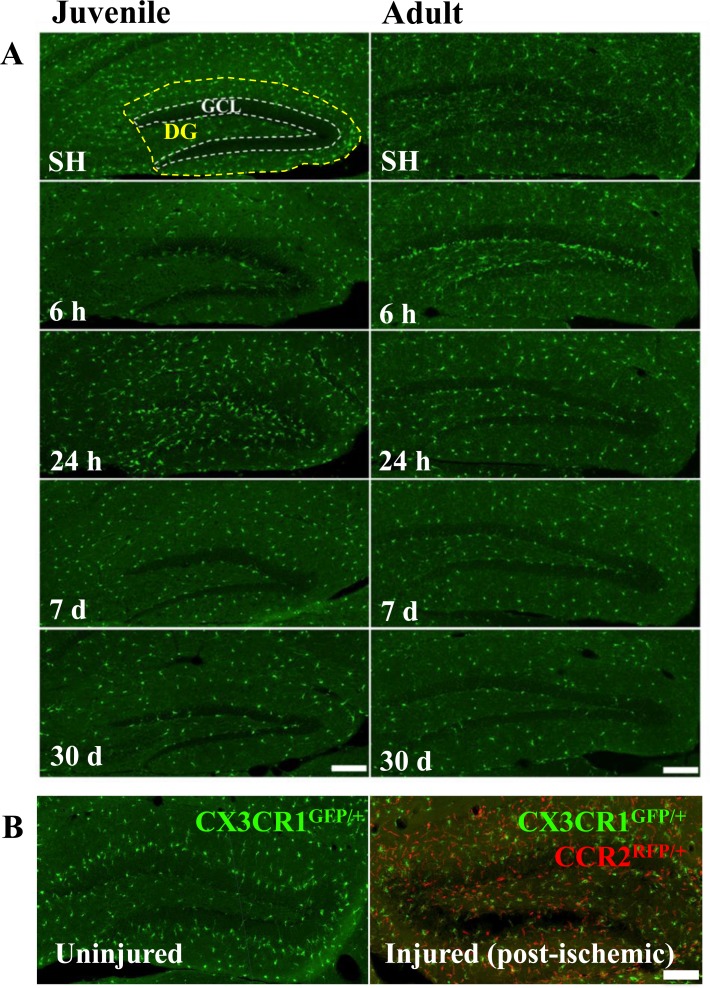
Irradiation did not recruit peripheral monocytes into the hippocampus No CCR2^RFP/+^-labeled cells were detected in the hippocampus at any of the time points after IR, neither in juvenile nor in adult brains. Scale bar, 150 μm (**A).** Unlike after IR, infiltrating monocyte-derived macrophages (CCR2^RFP/+^-labled cells, red) appeared in the injured hippocampus already 1 day after an ischemic insult (**B.**, right panel), in contrast to the uninjured hippocampus where only CX3CR1^GFP/+^-labeled cells (green) were detected (**B**, left panel). Scale bar, 100 μm. DG = dentate gyrus, GCL = granule cell layer.

Previous studies have suggested that high doses of IR increase blood-brain barrier permeability or even disruption [36]. We used CD31 and albumin staining to visualize blood vessels and possible vascular permeabilization, but we did not find any signs of extravascularization of albumin (not shown). Furthermore, there were no CCR2^RFP/+^-labeled cells in the parenchyma of the hippocampus (Figure 2A), or any other brain region (not shown), at any of the time points after IR, neither in juvenile nor in adult brains. Very rarely, an occasional CCR2^RFP/+^-labeled cell could be spotted inside a blood vessel, but never in extravascular locations. In contrast, after an ischemic insult, an injury where overt tissue injury and BBB disruption is known to occur, large numbers of CCR2^RFP/+^-labeled cells were recruited into the injured tissue within 1 day after the injury (Figure 2B) and numerous such cells could still be observed 1 month later (data not shown).

To determine whether monocytes and/or monocyte-derived macrophages may be activated in blood vessels or in other tissues and contribute to the inflammation in the brain, we generated mice with caspase-8-deficient myelomonocytic cells [37]. As expected, caspase-8 was deleted in mature myelomonocytic cells, but not in microglia (data not shown). Caspase-8 deletion changed neither microglia CCL2 mRNA expression (Supplementary Figure 1A), nor the IR-induced CCL2 protein increase (Supplementary Figure 1B), indicating that monocytes and/or monocyte-derived macrophages do not contribute to the inflammatory response in the hippocampus after IR.

### Microglia accumulation after IR was more pronounced and protracted in the juvenile hippocampus

**Figure 3:**
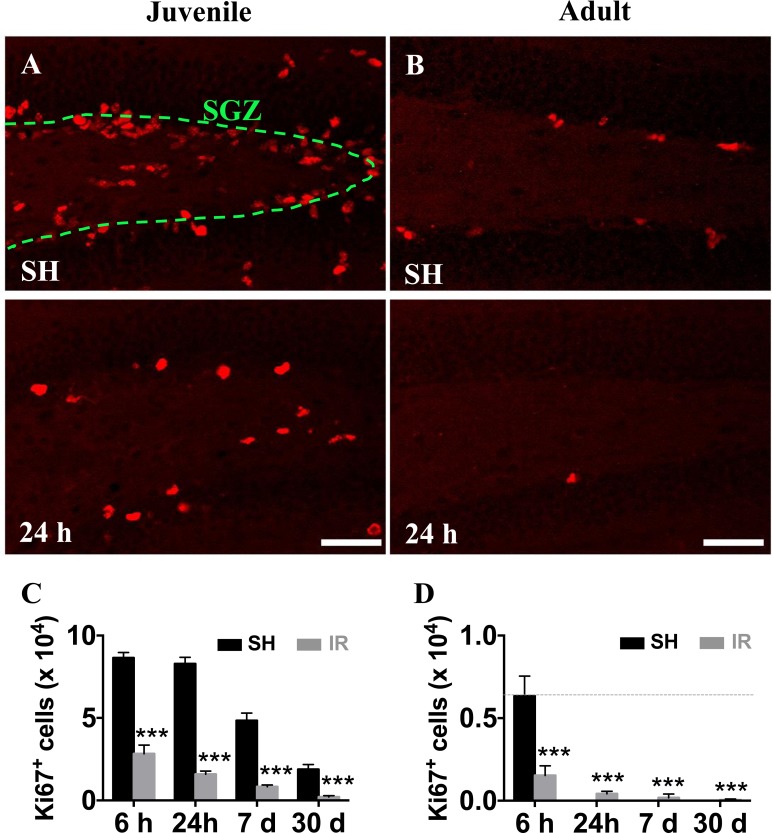
Irradiation reduced the number of hippocampal proliferating cells in both juvenile and adult brains Representative pictures showing cell proliferation (predominantly neural progenitor cells), as demonstrated by the numbers of Ki67-positive (red) cells, in the subgraular zone of the GCL in juvenile (**A**) and adult (**B**) hippocampi in sham (SH) controls and 24 h after IR. Ki67-positive cells decreased progressively after IR in both juvenile (**C**) and adult (**D**) brains. Scale bars, 50 μm. ***P < 0.001.

In agreement with our previous findings in rat [38] and mouse [13, 39] brains, we confirmed that a single dose of 8 Gy IR to the brain induced a significant - and progressive - reduction of proliferating (Ki67-positive) cells in the SGZ (Figure 3A-3D). Since monocyte-derived macrophages do not seem to contribute to the microglia/macrophage pool in the parenchyma after IR, neither in juvenile nor in adult mice, we characterized the response of the resident microglia in the affected GCL (including the neurogenic SGZ, where most of the cell death observed after IR occurs) and the entire DG.

**Figure 4:**
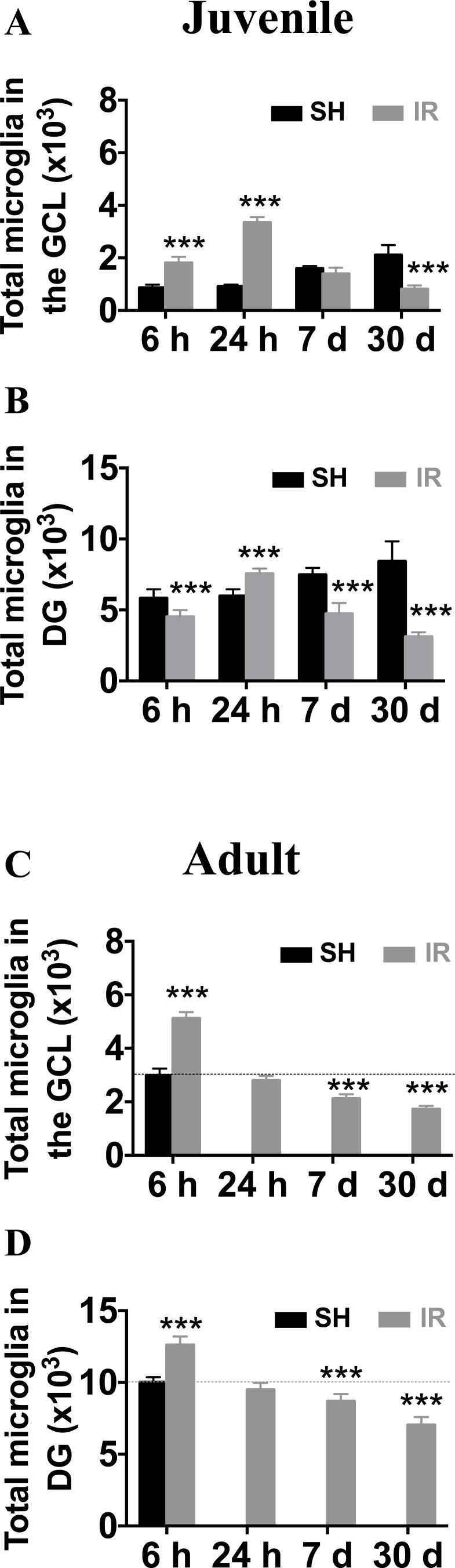
Irradiation induced a more pronounced and protracted microglia increase in the juvenile hippocampus The numbers of microglia were quantified in the GCL (including the SGZ) and the entire DG in sham controls (SH) or 6 h, 24 h, 7 days and 30 days after irradiation (IR). Data are shown as mean ± S.D., *n* = 6 for the sham groups, *n* = 10-11 for the irradiated groups. Asterisks indicate comparison between irradiated groups and the corresponding sham controls. For the juvenile brains, each time point is compared with a separate control group, taking into account possible developmental changes. ^***^P < 0.001.

In the juvenile brain, the microglia numbers in the GCL increased 6 h after IR (208 % of controls, from 873 ± 110 to 1,817 ± 228, *p* < 0.001), peaked at 24 h (366 % of controls, from 916 ± 72 to 3,353 ± 201, *p* < 0.001), returned to baseline at 7 days and decreased thereafter, to 39 % of controls (from 2,113 ± 376 to 820 ± 140, *p* < 0.001, Figure 4A). The changes in the entire DG (including the GCL) were different, with microglia numbers first decreased 6 h after IR (from 5,827 ± 626 to 4,515 ± 481, *p* < 0.001), somewhat increased at 24 h (from 5,982 ± 469 to 7,558 ± 359, *p* < 0.001) and decreased thereafter to 63 % lower than sham controls 30 days after IR (from 8,433 ± 1,398 to 3,122 ± 309, *p* < 0.001, Figure 4B).

In adult brains (P 90), however, the microglia numbers in the GCL peaked already 6 h after IR (171 % of controls, from 2,983 ± 260 to 5,116 ± 240, *p* < 0.001) and returned to baseline within 24 h. Thirty days after IR, the average number was 42 % lower than that of sham controls (from 2,983 ± 260 to 1,725 ± 126, *p* < 0.001, Figure 4C). In the entire DG, the time course was similar to that of the GCL, but the increase at 6 h after IR was only marginal (126 % of controls, from 10,040 ± 331 to 12,610 ± 587, *p* < 0.001) and the decrease was 30 % at 30 days after IR (from 10,040 ± 331 to 7,048 ± 543, *p* < 0.001, Figure 4D).

In summary, both juvenile and adult brains displayed a transient increase in the number of microglia, mainly in the GCL/SGZ, where cell death occurs, followed by a gradual and pronounced decrease over time, at least up to 30 days after IR. The response was quicker, but also less pronounced, in the adult brain.

### Microglia proliferation contributed to the increased numbers after IR in the juvenile, but not in the adult, hippocampus

**Figure 5:**
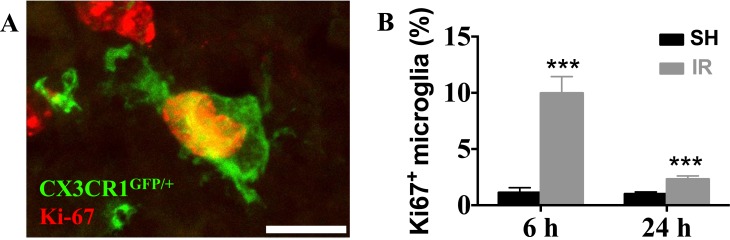
Irradiation induced microglial proliferation only in the juvenile brain A representative microphotograph showing co-localization of microglia (CX3CR1^GFP/+^, green) and Ki67 (red) in the juvenile DG after IR, indicating proliferating microglia (**A**) In juvenile brains, microglial proliferation was increased 6 h and 1 d after IR (**B**) No Ki67-positive microglia could be detected in the DG of adult brains.

The number of proliferating (Ki67-positive) microglia (Figure 5A) in the juvenile DG increased 809 % 6 h after IR (from 1.1 ± 0.4 % to 10.0 ± 1.5 %, *p* < 0.001) and 130 % 24 h after IR (from 1.0 ± 0.2 % to 2.3 ± 0.3 %, *p* < 0.001) (Figure 5B). At later time points, 7 and 30 d after IR, no Ki67-positive microglia were detected, neither in controls, nor after IR. In the adult DG, however, no Ki67-positive microglia could be detected in any time point, neither in controls, nor after IR (not shown). Hence, the more pronounced increase in microglia numbers after IR in the juvenile, but not in the adult, hippocampi could partly be attributed to proliferation.

### Microglia cell death contributed to the reduced numbers after IR

**Figure 6:**
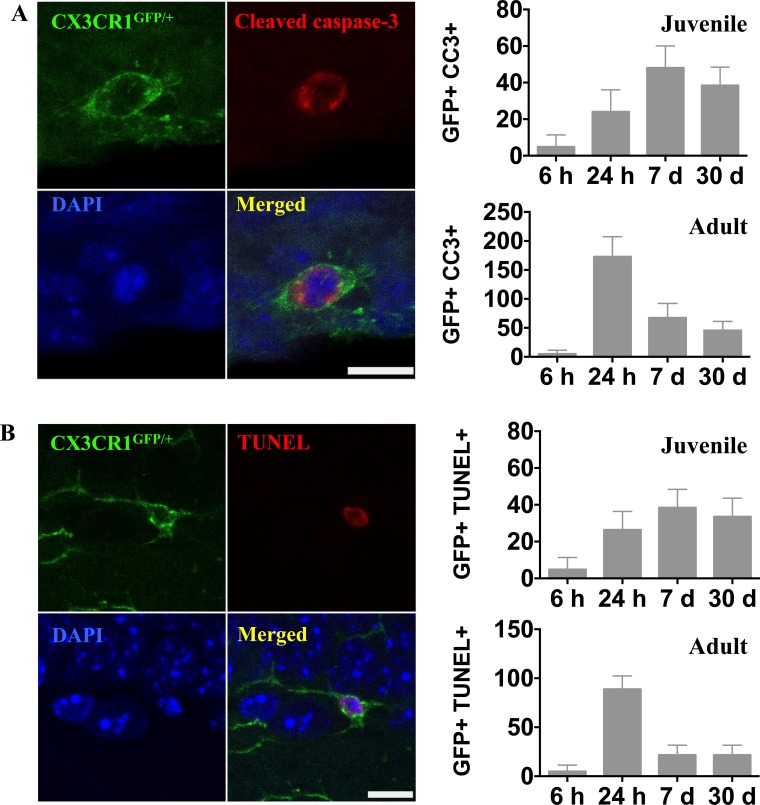
Irradiation induced microglial cell death **A.** Representative microphotographs showing co-localization of microglia (CX3CR1^GFP/+^, green) and activated caspase-3 (red) in the juvenile DG after IR. The numbers of activated caspase-3-positive microglia were quantified in the entire DG in sham controls (SH) or 6 h, 24 h, 7 days and 30 days after irradiation (IR). Data are shown as mean ± S.D., *n* = 6 for the sham groups, *n* = 10-11 for the irradiated groups. Scale bar, 10 μm. **B.** Representative microphotographs showing triple staining of CX3CR1^GFP/+^, TUNEL (red) and DAPI (blue). Only microglia with a clearly TUNEL-positive nucleus were counted, distinguishing them from microglia with engulfed chromatin fragments from phagocytosed cells. The numbers of TUNEL-positive microglia were quantified in the entire DG in sham controls (SH) or 6 h, 24 h, 7 days and 30 days after irradiation (IR). Data are shown as mean ± S.D., *n* = 6 for the sham groups, *n* = 10-11 for the irradiated groups. Scale bar, 10 μm.

We stained for activated caspase-3 and DNA strand breaks (TUNEL) to investigate microglial cell death after IR. In contrast to the rapid onset of microglial activation and proliferation, caspase-3 activation and chromatin fragmentation following IR, indicating cell death, was delayed. In juvenile brains, caspase-3-positive microglia were detected (4.8 ± 6.6) in the DG 6 h after IR. The number of dying microglia had increased by 24 h (24.0 ± 12.0), reached a peak by 7 days (48.0 ± 12.0), and decreased to 38.4 ± 10.0 30 days after IR (Figure 6A). In adult brains, however, microglia cell death in the DG peaked already 24 h after IR (172.8 ± 34.6) and decreased thereafter to 67.2 ± 24.9 at 7 days. Thirty days after IR, microglia cell death could still be detected (45.6 ± 15.7) (Figure 6A).

The appearance of TUNEL-positive microglia followed a very similar time course as that of active caspase-3, and the numbers were in the same range (Figure 6B). Only microglia with a clearly TUNEL-positive nucleus were counted, distinguishing them from microglia with engulfed chromatin fragments from other cells. Since these two essentially different methods for detection of cell death yielded very similar results, we interpret this as reflective of caspase-dependent microglial apoptosis.

### Microglial activation after IR was less pronounced and more transient in the adult hippocampus

**Figure 7:**
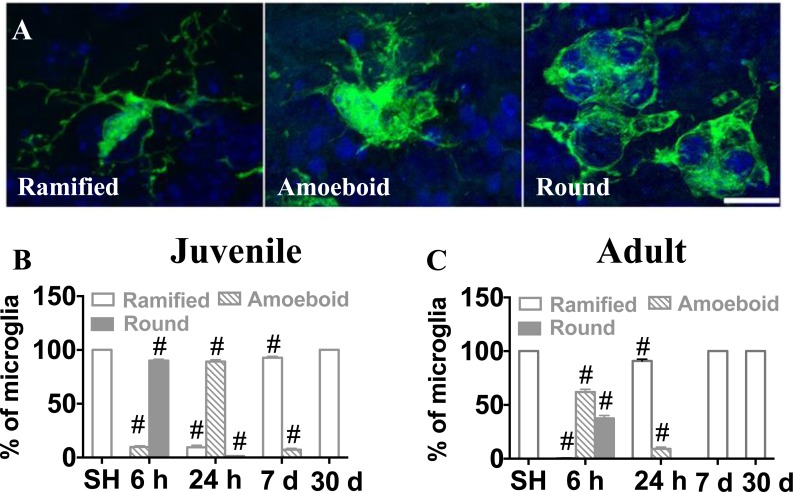
Microglia in the SGZ underwent morphological changes after irradiation Microglia were classified into ramified, amoeboid and round phenotypes based on their morphological characteristics (**A**) The percentages of microglia with the different phenotypes of the total number of microglia in sham-irradiated (SH) or 6 h, 24 h, 7 days and 30 days after IR in juvenile (**B**) and adult (**C**) brains were assessed. Sham controls for the juvenile brains collectively represent P10, P11, P17 and P40, since the results were identical. Data are shown as mean ± S.D., *n* = 6 for the sham groups, 10-11 for the irradiated groups.Single hash tags indicate comparison between irradiated groups and the corresponding sham controls. ^#^*P* < 0.001.

**Figure 8:**
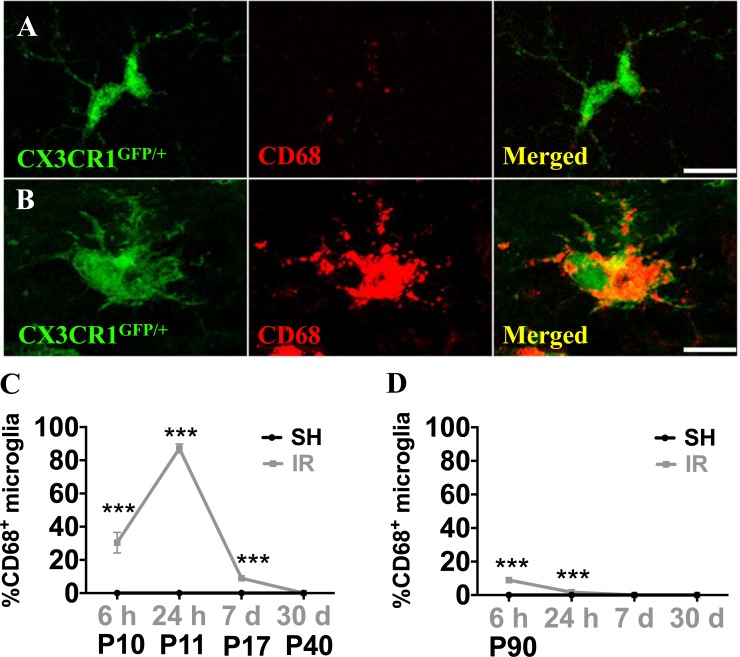
Microglia in the SGZ expressed CD68 after irradiation CD68 expression was very low in ramified microglia in sham controls, but increased strongly in some microglia after IR (**A**, **B**) The numbers of microglia with high CD68 staining (as in B) were counted. Percentages of microglia with high CD68 expression over the total number of microglia in the SGZ after sham irradiation (SH) or 6 h, 1 day, 1 week and 1 month after irradiation (IR) in juvenile (**C**)and adult (**D**) brains. Data are shown as mean ± S.D., *n* = 6 for the sham groups, 10-11 for the irradiated groups. Asterisks indicate comparison between irradiated groups and the corresponding sham controls. ***P < 0.001. Scale bars, 10 μm.

Microglia undergo morphological changes in response to various insults, including when they phagocytose dead cells. In this study, we counted microglia based on their morphological characteristics. Microglia were classified into three morphological phenotypes, ramified, amoeboid or round (Figure 7A), and the numbers of each phenotype were quantified in the GCL. In sham-treated animals, all microglial cells were ramified. In the juvenile brains, microglia underwent rapid structural changes upon IR. No ramified microglia were observed 6 h after IR, the majority (90 %) exhibited a round morphology, indicating phagocytosis. Round microglia were markedly decreased after 24 h and had disappeared after 7 days. Amoeboid microglia peaked after 24 h and gradually decreased until 7 days after IR. Concurrently, a small portion of microglia exhibited ramified morphology again after 24 h and the number gradually increased thereafter until all of them were ramified again after 30 days (Figure 7B). In adult brains, morphological microglia changes were transient. As in the juvenile brains, most microglia were round or amoeboid 6 h after IR, but in the adult brains most of them had resumed a ramified morphology already after 24 h, by when only 9 % were amoeboid. After 7 days and 30 days, all microglia were ramified, as in sham controls (Figure 7C). CD68 expression is increased in morphologically activated microglia [40] (Figure 8A, 8B) and in our model it correlated closely with the appearance of amoeboid microglia. However, in juvenile hippocampi almost 90 % of the microglia were CD68-positive in the SGZ at the peak of activation (Figure 8C), compared with only 9 % in adult hippocampi (Figure 8D). Taken together, both the morphological changes and the CD68 expression demonstrate a less pronounced and more transient activation in adult brains.

### Microglial gene and surface marker expression revealed a more pro-inflammatory profile in juvenile hippocampi

**Figure 9:**
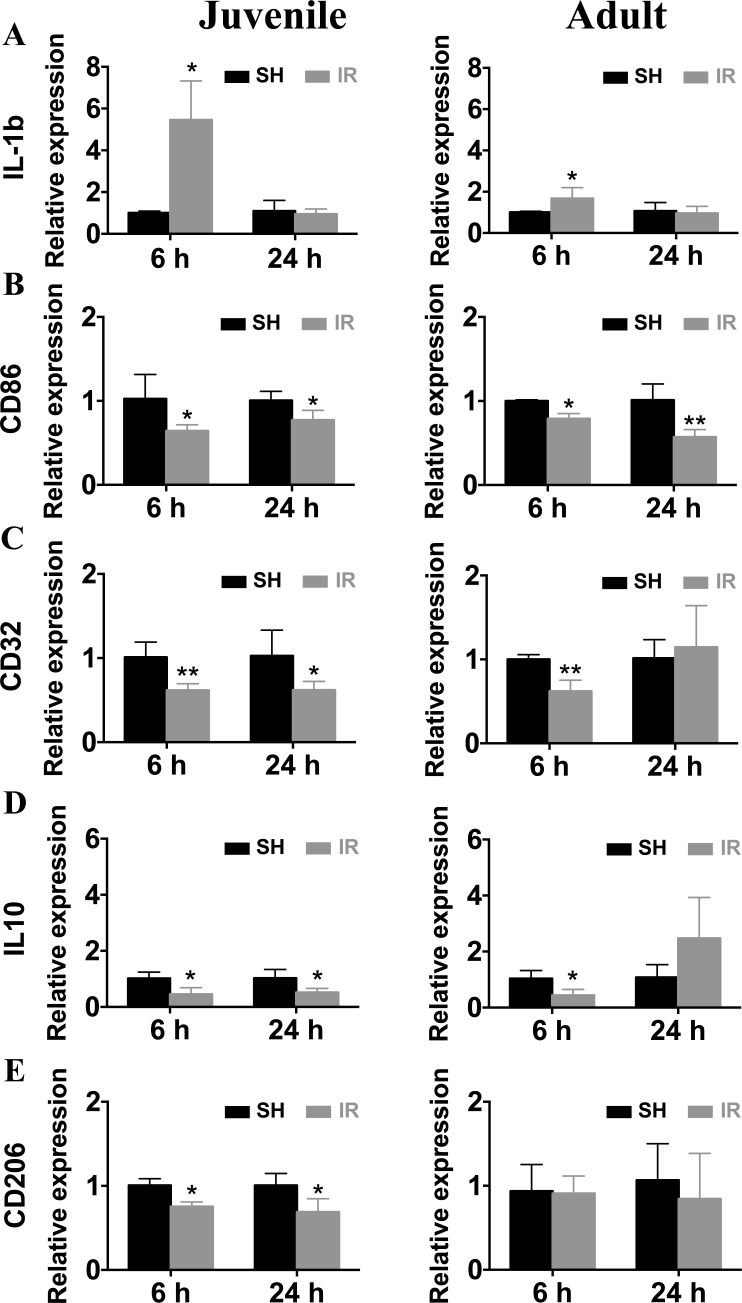
Irradiation induced changes in the gene expression of microglial markers Microglia from juvenile and adult hippocampi expressed a significantly higher IL-1β mRNA levels 6 h after IR compared to sham controls (SH) (**A**) The mRNA levels of CD86 (**B**), CD32 (**C**), IL-10 (**D**) and CD206 (**E**) were significantly reduced after irradiation (IR), and the reduction was more pronounced and protracted in microglia from juvenile brains compared with their adult counterparts. Data are shown as mean ± S.D., *n* = 3 for the sham groups, 5-6 for the irradiated groups. Asterisks indicate comparison between irradiated groups and the corresponding sham controls. *P < 0.05, **P < 0.01.

Since microglia responses, both quantitatively and phenotypically, were more pronounced 6 h and 24 h after IR in both juvenile and adult hippocampi, we isolated microglia from hippocampi at those time points and analyzed microglia gene expression *ex vivo*. Using real time PCR, we found that IL-1β levels were increased 6 h after IR and returned to sham levels by 24 h in both juvenile and adult microglia (Figure 9A). The increase in juvenile microglia was much more pronounced, though (446 % compared with 67 %). By contrast, the levels of CD86 (Figure 9B), CD32 (Figure 9C), IL-10 (Figure 9D) and CD206 (Figure 9E) were all decreased at both time points in the juvenile microglia. The changes in adult hippocampal microglia, however, were different; while CD86 expression was decreased at both time points (Figure 9B), CD32 (Figure 9C) and IL-10 (Figure 9D) expression were decreased only 6 h after IR, and CD206 (Figure 9E) expression was unchanged.

**Figure 10:**
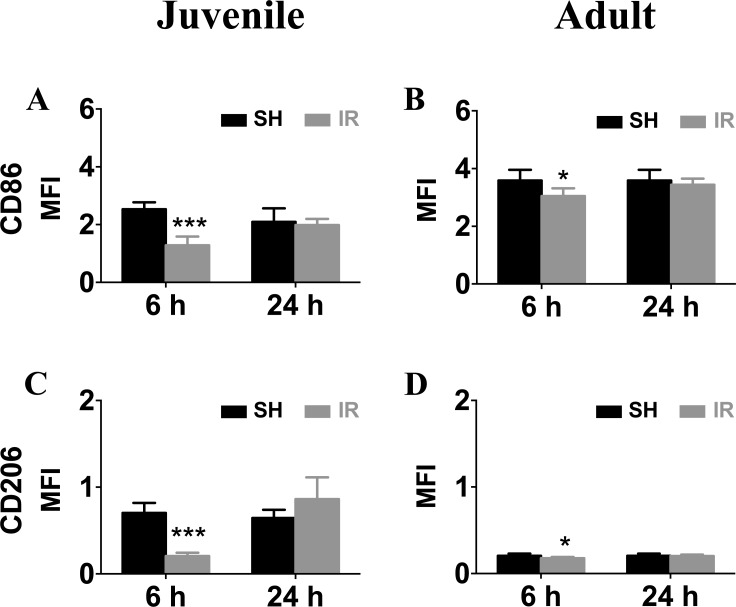
Irradiation downregulated microglia surface expression of CD86 and CD206 Representative histograms of CD86 and CD206 surface levels on microglia isolated from sham control (SH) or irradiated (IR) hippocampi from juvenile (left panel) and adult brains (right panel). Surface mean fluorescence intensity (MFI) for CD86 (**A**) and CD206 (**B**) Data are shown as mean ± S.D., *n* = 3 for the sham groups, 5-6 for the irradiated groups. Asterisks indicate comparison between irradiated groups and the corresponding sham controls. *P < 0.05, ***P < 0.001.

We also performed FACS to analyze microglia surface marker expression. In accordance with the gene expression levels, surface mean fluorescence intensities (MFI) of CD86 (Figure 10A, 10B) and CD206 (Figure 10C, 10D) were down-regulated 6 h after IR in both juvenile and adult hippocampal microglia, but the decrease was only marginal in adult microglia (Figure 10B, 10D). Taken together, the overall expression profile was more pro-inflammatory by nature in microglia isolated from irradiated juvenile brains than from adult brains.

### Long-lasting elevation of CCL2 and IL-1β expression in both juvenile and adult hippocampi after IR

**Figure 11:**
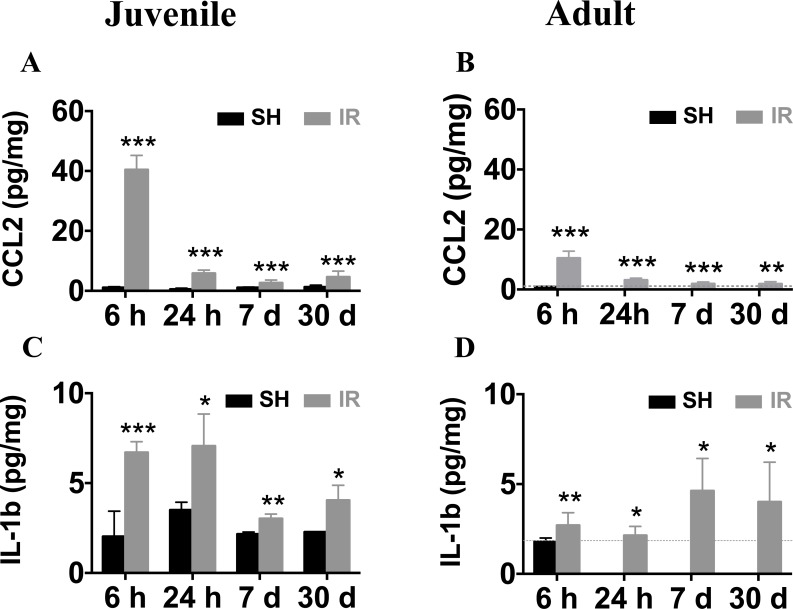
Irradiation induced persistent elevation of CCL2 and IL-1β expression in the hippocampus Very low levels of CCL2 were detected in sham controls (SH). Irradiation (IR) increased CCL2 expression in both juvenile (**A**) and adult (**B**) hippocampi, displaying a peak 6 h after IR, and still being elevated 30 days after IR. The expression levels of IL-1β were also persistently elevated for at least 30 days both in juvenile (**C**) and adult (**D**) brains. Data are shown as mean ± S.D., *n* = 6 for the sham groups, 10-11 for the irradiated groups. Asterisks indicate comparison between irradiated groups and the corresponding sham controls. For the juvenile brains, each time point is compared with a separate control group, taking into account possible developmental changes. *P < 0.05, **P < 0.01, ***P < 0.001.

CCL2 is one of the most strongly upregulated cytokines or chemokines after IR in both juvenile and adult rodent brains [23, 25, 41]. In addition, IL-1β is a key pro-inflammatory cytokine that drives neuroinflammatory processes in a variety of neurodegenerative diseases and exerts neurotoxic effects [42–44]. We determined the time courses of CCL2 and IL-1β expression in the juvenile and adult mouse hippocampus after IR by using ELISA, demonstrating that the CCL2 expression peaked 6 h after IR in both juvenile (Figure 11A) and adult (Figure 11B) brains, with 4-fold higher levels in the juvenile hippocampus (40.4 ± 1.4 vs. 10.4 ± 0.8 pg/mg). The expression of IL-1β at 6 h after IR was more than twice as high in the juvenile hippocampus when compared with its adult counterpart (Figure 11C-11D, 6.7 ± 0.6 vs. 2.7 ± 0.7 pg/mg). Importantly, the levels remained increased at all the time points investigated after IR in the hippocampus, i.e. for at least 30 days, both in juvenile and adult brains (Figure 11A-11D), suggesting that IR induced a chronic inflammation.

## DISCUSSION

The main findings in this study were:

1. The absence of monocyte-derived macrophages in brain tissue after IR
2. The more pronounced, protracted and pro-inflammatory reaction in juvenile brains.
3. The long-lasting loss of microglia after IR.
4. The chronic nature of the resulting neuroinflammation.

The absence of infiltrating blood-borne, monocyte-derived macrophages after IR taking on a microglial phenotype in the irradiated tissue came as a surprise, given the substantial contribution of these cells to the macrophage population in injured areas of the brain after for example an ischemic insult (Figure 3B) [31, 45]. We demonstrate this for the hippocampus, but it was the case in the entire brain (not shown). It has been shown that CCR2 expression (and therefore RFP in this case) is downregulated once monocytes infiltrate the CNS and differentiate into macrophages [46], so we used an anti-RFP antibody to amplify the RFP signal. Occasional RFP-positive cells could be detected inside blood vessels, but never in the parenchyma (not shown). It is conceivable that monocytes or monocyte-derived macrophages may be activated in the blood vessels or in other tissues and contribute to the inflammation in the brain after IR, but our experiments using caspase-8-deficient monocytes could not find any evidence for this. Using the same strain of mice Morganti *et al*. could detect blood-derived macrophages in the normal brain parenchyma using FACS, but these numbers were not altered by 10 Gy cranial radiation in adult animals [47]. The carefully executed study of Lee *et al*. [25], also administering a single dose of 10 Gy to the brains of adult mice, obtained results consistent with both ours and those of Morganti *et al*.. Bone marrow-derived cells were identified using three different methods, by elevated CD45 expression, by labeling of circulating monocytes with dextran-coated ultra-small super-paramagnetic iron oxide particles, or by EGFP expression in bone marrow chimeras, and in no case did they find evidence of IR-induced infiltration of bone marrow-derived cells in the brain [25]. CCL2, previously called monocyte chemoattractant protein-1, is known for its role in regulating BBB permeability by modulating tight junction proteins in endothelial cells [48] and for recruiting neutrophils and macrophages to injury sites under multiple pathological conditions [49], thus playing an important role in regulating the inflammatory response. Increased CCL2 has been associated with decreased neurogenesis [50], but this was apparently not sufficient to recruit monocytes into the brain tissue after IR. We did not find evidence of increased BBB permeability after 8 Gy, as judged by extravascularization of albumin. Using a much higher dose (20 Gy single dose), Yuan et al. observed increased BBB permeability and adherent leukocytes in pial vessels, but not in animals subjected to a lower IR dose (5 Gy) [36]. Hence, unless very high doses are used, causing radiation necrosis and overt tissue injury, there appears to be very limited BBB permeabilization or leukocyte infiltration after IR.

The more pronounced, protracted and pro-inflammatory reaction in juvenile brains is consistent both with the higher levels of cell death and with the increased vulnerability of young brains to ionizing radiation [1, 19, 38], but it remains to be shown if the pronounced neuroinflammation in juvenile brains simply is a result of the greater numbers of dying progenitor cells after IR [22, 38], or if the more pronounced inflammatory reaction itself contributes to and explains the more devastating consequences of cranial radiotherapy in children. These two explanations are of course not mutually exclusive. Our results, demonstrating that microglia isolated from irradiated juvenile brains display a more pro-inflammatory profile than those from adult brains, e. g. more reduced CD206 and more increased IL-1β expression, indicate the presence of cell-intrinsic properties that would support the hypothesis that the juvenile brain responds more vigorously to an insult. The finding that microglia proliferated after IR only in the juvenile brain also supports this notion.

The long-lasting loss of microglia observed after IR likely has functional consequences if the brain is subjected to subsequent, repeated insults. It remains to be investigated how juvenile and adult brains respond to fractionated IR in this respect compared with a single dose. The IR dose used in this study (8 Gy) is equivalent to 18 Gy when delivered in repeated 2 Gy fractions, according to the linear-quadratic model [51] using an α/β ratio of 3 for late effects in normal brain tissue, and represents a clinically relevant, moderate radiation dose. The more pronounced loss of microglia in juvenile brains may, at least partly, be explained by the higher levels of proliferation observed in juvenile brains. No proliferating microglia could be detected in the adult brains at any time point, neither in controls, nor after IR, but the baseline level was around 1 % in the juvenile brains, in accordance with earlier publications demonstrating a wave of microglia proliferation during normal development in the postnatal day 9 mouse brain [52]. The reason for the increased numbers of microglia in the GCL after IR also in the adult brains, in the absence of proliferation, was migration to the SGZ, where progenitor cell death occurs, from the neighboring molecular layer and the hilus. This occurred in both age groups and was apparent from reduced numbers in these neighboring regions corresponding approximately to the increase in the GCL (not shown), and can be understood when comparing the numbers in the GCL (Figure 5A, 5C) with those of the entire DG (Figure 5B, 5D). Already 6 h after IR, microglia proliferation had increased to 10.0 % in juvenile brains and was still increased after 24 h (2.3 %). Proliferating cells are particularly susceptible to IR and this is likely one important reason for the greater relative loss of microglia in the juvenile brains. We previously found that the number of microglia was decreased 7 days after IR in juvenile (P9 and P21) mice [24] and in both juvenile and adult rat hippocampi 4 weeks after IR [22, 38]. Morganti *et al*., however, used the same reporter strain of mice and found that 28 days after 10 Gy cranial IR to adult mice the percentage of microglia had returned to sham levels [47]. They did not use histological quantification according to stereological principles, though. An elegantly designed study using comprehensive transcriptional profiling of microglia 1 month after IR in mice, demonstrated aging-like changes in the transcriptome of irradiated microglia, including that expression of the colony-stimulating factor 1 receptor (CSF1R) gene was inversely correlated with radiation treatment [53]. Given that CSF1R is necessary for microglia viability [54], it is tempting to speculate that IR induced microglial loss at least partly by inhibiting CSF1R signaling. The numbers of dying microglia in the DG at any given time point may seem small, ranging from approximately 25-50 in the juvenile and 50-170 in the adult, but considering that the duration of cell death is estimated to be in the range of 4-24 h [55, 56], the accumulating number of microglia over this long period of time, from 24 to 30 days after IR, is approximately equivalent to the total loss of cells observed 30 days after IR. We found that in the entire DG only 27 % of the microglia remained in the juvenile brain 30 days after IR, compared with 70 % in the adult brains. This dramatic loss of microglia, a cell type crucial for surveillance, turnover of cellular debris and regulation of inflammation, may also explain why the juvenile, still growing brain suffers more devastating consequences after cranial radiation therapy.

The chronic nature of the resulting neuroinflammation was demonstrated by the increased levels of both CCL2 and IL-1β in both juvenile and adult hippocampi at all time points after IR. This has previously been implicated by Monje *et al*., demonstrating CD68-positive microglia in the hippocampus 2 months after 10 Gy to adult rat brains [17]. Using multiplex analysis of 24 or more chemokines and cytokines (Luminex) in brain tissue after 8 Gy IR to juvenile rats, we found that the levels of CCL2, for example, increased 6 h and 12 h after IR, but had returned to baseline levels by 24 h or 48 h [23]. However, we found that while the Luminex technology allowed simultaneous quantification of 24 analytes, the sensitivity for each individual agent analyzed was greater if we used ELISA. Lee *et al*. [25] had the same experience, so we decided to quantify CCL2 and IL-1β using ELISA, revealing significantly elevated levels after IR at all time points analyzed. Some of the proposed roles for CCL2 have been mentioned above. IL-1β is able to increase BBB permeability by suppressing astrocytic sonic hedgehog production and abolishing the protective effect of astrocytes on BBB integrity [57]. Moreover, increased IL-1β negatively impacts hippocampal neurogenesis by creating an inflammatory milieu that skews neurogenesis toward gliogenesis [58] and by directly interacting with neural progenitor cells to cause cell cycle arrest [59]. A broad range of cells can express CCL2 and IL-1β, including microglia, astrocytes and endothelial cells [60–62], and it remains to be determined which specific cell type(s) that are responsible for the initial and chronic production, respectively. For IR-induced brain injury, as well as all other types of brain injury where there is a component of chronic inflammation, this is an attractive target for neuroprotective strategies. If the transition from acute to chronic inflammation can be abolished, the functional outcomes, and thereby the quality of life, of the increasing number of patients surviving their brain tumor, can be improved.

## MATERIALS AND METHODS

### Animals

C57BL/6 mice were obtained from Charles River (Sulzfeld, Germany). CX3CR1^GFP/+^CCR2^RFP/+^ double heterozygous mice were generated by crossbreeding CX3CR1^GFP/GFP^ with CCR2^RFP/RFP^ transgenic mice (Jackson Laboratory, Bar Harbor, ME, U.S). Caspase-8^fl/fl^ C57BL/6 and LysMCre^+/−^ Caspase-8^fl/fl^ C57BL/6 mice were kindly provided by Jose-Luis Venero (University of Seville, Seville, Spain). Cross-breeding between these two strains generated 50 % Casp8^fl/fl^ LysMCre^+/−^ and 50 % Casp8^fl/fl^ LysMCre^−/−^. The genotype was confirmed for each animal using protocols provided by the suppliers. CX3CR1^GFP/+^CCR2^RFP/+^ animals of both sexes at postnatal day (P) 10 or 90 were used in this study. Casp8^fl/fl^ LysMCre^+/−^ and Casp8^fl/fl^ LysMCre^−/−^ mice were used at P21. Mice in the same litter were randomly assigned to IR or sham treatment, and housed in a 12-h light/dark cycle with food and water available *ad libitum*. All experiments were conducted according to the institutional approved protocols and were approved by the Karolinska Institutet ethical committee (application number N9/12).

### Cranial irradiation

For the IR procedure, animals were put onto a carved Styrofoam bed adjusted for body size with a source to sample distance of 50 cm in a cabinet X-ray irradiator (XRAD320, Precision X-Ray, North Branford, CT, U.S.) and anesthetized with isoflurane (5 % for induction, 2 % for maintenance) during the procedure. The whole brain was covered in a radiation field of 2 × 2 cm and the dose rate was 0.72 Gy/min at 320 kV, 12.5 mA. A single dose of 8 Gy was delivered, yielding a time for the IR procedure of 11 minutes and 3 seconds. The total procedure, including induction of anesthesia, for each animal took around 13 minutes. Sham control animals were anesthetized but not subjected to IR. After IR, the animals were returned to their cages, with or without dams depending on the age of the animals, until the experimental endpoints. Using the linear-quadratic model [51] and an α/β ratio of 3 for late effects in normal brain tissue, the acute exposure of 8 Gy is equivalent to 18 Gy when delivered in repeated 2 Gy fractions, thereby representing a clinically relevant, moderate radiation dose. For example, in the most recent medulloblastoma protocol, PNET5, patients are treated with 18 or 23.4 Gy to the whole brain and spinal cord, plus a 30.6 Gy boost to the primary tumor site.

### Induction of hypoxia-ischemia

Unilateral HI was induced in P9 CX3CR1^GFP/+^CCR2^RFP/+^ mice according to the Vannucci model, adapted for mice [31]. Briefly, the mice were anesthetized with isoflurane (5.0 % for induction and 1.5- 3.0 % for maintenance) in a mixture of normal air and oxygen (1: 1). The right common carotid artery of each pup was ligated with Prolene sutures (6.0). After the wound was sutured the mice were returned to their dams, allowed to recover for 2 h, and then placed in an incubator perfused with a humidified gas mixture (10% oxygen in nitrogen) at 36°C for 50 min. Control animals did not undergo surgery or hypoxia.

### Tissue preparation

At 6 hours, 24 hours, 7 days or 30 days after IR, animals were deeply anaesthetized with an overdose of sodium pentobarbital (50 mg/kg, Pentothal, Electra-box Pharma, Stockholm, Sweden), and transcardially perfused with ice-cold phosphate-buffered saline (PBS). Mice were decapitated, the hippocampus of one hemisphere was quickly dissected out and snap frozen in dry ice and stored at −80°C. The other hemisphere was immersed into 4 % paraformaldehyde (PFA) for 3 days at 4°C before being transferred into gradient sucrose solutions (10 %, 20 % and 30 % sucrose in 0.1 M phosphate buffer). When fully saturated, the brains were frozen fixed with a cryo-gel (Tissue-Tec O.C.T compound) onto a steel block on a bed of dry ice and sagittally cut into 25-μm sections using a sliding microtome (Leica SM 2000R, Leica Microsystems, Nussloch, Germany). Collection of sections in a series of 12 tubes containing tissue cryoprotectant solution (25 % ethylene glycol and 25 % glycerin in 0.1 M phosphate buffer) started when reaching a level with the typical C-shaped structure of the hippocampus, typically ending up with 4 sections containing the hippocampus from juvenile brains (P 10, p 11, p 17) and 5 sections from adult brains (P 40, p 90). The sections were stored at 4°C until further use.

### Protein extraction and ELISA

Each hippocampus was homogenized in 5 volumes extraction buffer (50 mM Tris-HCl, pH 7.5, 100 mM NaCl, 2 mM EDTA-Na, 1 % Triton X-100, and protease inhibitor cocktail tablet (11836170001, Roche Diagnostics, Basel, Switzerland)) by sonication. After centrifugation at 15,000 x g for 10 minutes at 4°C, the supernatant was aliquoted and stored at −80°C. Protein concentration was determined by using a BCA assay kit (Pierce, IL, U.S), and CCL2 and IL-1β expression levels were quantified using ELISA kits in accordance with the instructions of the manufacturer (mouse JE/CCL2 and mouse IL-1 beta kits, R&D systems, MN, U.S).

### Immunofluorescent staining and cell counting

The sections were rinsed with Tris-buffered saline (TBS, 50 mM Tris-HCl in 150 mM NaCl, pH 7.5). To prevent non-specific binding, the sections were incubated in blocking solution (3 % donkey serum in TBS with 0.1 % Triton X-100) for 1 h at room temperature (RT). After blocking, the sections were incubated overnight at 4°C with primary antibodies diluted in blocking solution (rabbit anti-Iba-1 1:1,000, Wako; mouse anti-NeuN 1:200, MAB377, Chemicon; rabbit anti-GFAP 1:1,000, AB5804, Millipore; rabbit anti-RFP 1:1,000, 632496, Clonetech; rabbit anti-activated caspase-3 1:500, 9579S, Cell Signaling). On the second day, after rinsing with TBS three times, the sections were incubated with appropriate fluorophore-conjugated secondary antibodies for 1 h at RT. After washing, the sections were mounted using Prolong anti-fade mountant with DAPI (P36935, Invitrogen). We performed CD31 and albumin staining to check blood-brain barrier permeability. The sections were rinsed with TBS, followed by the incubation of primary antibodies (rabbit anti-CD31, 1:500, BD bioscience; goat anti-mouse albumin, 1:10,000, Abcam) at 4°C overnight, then the sections were incubated with appropriate fluorophore-conjugated secondary antibodies for 1 h at RT and mounted. Click-iT plus TUNEL assay kit was used to detect cellular DNA strand breaks according to the manufacturer's instructions (C10618, Invitrogen). Negative controls were subjected to the same staining procedures but without the primary antibodies.

Counting of GFP-labeled microglia and RFP-labeled monocytes was performed on maximum intensity projections from z-series (10-12 slices per image) obtained at 20 X magnification using a confocal laser-scanning microscope. The cell numbers in the granule cell layer (GCL, including the neurogenic subgranular zone (SGZ)) and the entire dentate gyrus (DG) were assessed and the corresponding volumes of each region were measured. Based on the morphological differences, microglia were classified into ramified, amoeboid and round phenotypes. The percentage of the different morphological subtypes of microglia was based on analysis of the cells in each animal in the GCL.

### Microglia isolation and flow cytometry

Hippocampi from C57BL/6 mice were quickly dissected after perfusion with ice-cold PBS 6 hours or 24 hours after IR, cut into 1mm^3^ pieces and enzymatically digested in 2.5 ml HBSS medium containing 15 mM HEPES, 2 mM EDTA, 5 mg/ml BSA, 10 mU/ml DNase I and 100 mU/ml papain for 20 min at 37°C with constant agitation (180/min). Further processing was performed at 4°C. After removing tissue debris by passing cell suspension through a 40 μm cell strainer, cells were incubated with CD11b magnetic beads for 20 min, CD11b+ cells were separated and collected in a magnetic field using MS columns (Miltenyi Biotec, Germany). The amounts of magnetic beads were calculated based on the number of cells obtained after debris removal, following the manufacturer's instructions. Microglia were incubated with antibodies against CD86, and CD206 (eBiosciences) for 30 min. Cells were then washed with PBS and re-suspended in flow buffer. Expression of surface antigens was analyzed using a Beckman Coulter flow cytometer (Brea, CA, USA). A minimum of 6,000 events was recorded for each sample and analyzed using Kaluza^®^ software (Brea, CA, USA).

### Caspase-8 deletion in myelomonocytic cells

Microglia were isolated from the whole brain of sham and irradiated mice as described above. For the isolation of mononuclear cells, the blood from LysMCre^−/−^ Caspase-8^fl/fl^ and LysMCre^+/−^ Caspase-8^fl/fl^ C57BL/6 mice was obtained by cardiac puncture and diluted with an equal amount of PBS plus 2% fetal bovine serum (07905, Stem cell technologies, Vancouver, BC, Canada). The mixture was gently overlaid on top of Lymphoprep (07801, Stem cell technologies, Vancouver, BC, Canada) and centrifuged at 800 × g for 20 minutes at room temperature with brake off. Mononuclear cells were then transferred from the interface between plasma and Lymphoprep layers and washed twice before DNA extraction.

PCR analysis using three primers was applied for evaluating caspase-8 gene deletion efficiency, 5′- ATAATTCCCCCAAATCCTCGCATC-3′ (S for the wild-type, floxed, and deleted allele), 5′- GGCTCACTCCCAGGGCTTCCT-3′ (AS for the wild-type, and floxed allele) and 5′- GCTACAGTG ATGGTTTGTACATGG-3′ (AS for the deleted allele) (Sigma-Aldrich, St Louis, MO, USA).

### qRT-PCR analyses

Total RNA was isolated from *ex vivo* microglia from CX3CR1^GFP/+^CCR2^RFP/+^, LysMCre^−/−^ Caspase-8^fl/fl^ and LysMCre^+/−^ Caspase-8^fl/fl^ C57BL/6 mice using the RNeasy Micro Kit (QIAGEN) according to the manufacturer's instructions. The following primers were used *(5' to 3′ S/AS): CD32, 5′-AATCCTGCCGTTCCTACTGATC-3′/5'-GTGTCACCGTGTCTTCCTTGAG-3′; CD86, 5′-GACCGTTGTGTGTGTTCTGG-3′/5'-GATGAGCAGCATCACAAGGA-3′; CD206, 5′-CAAGGAAGGTTGGCATTTGT-3′/5'-CCTTTCAGTC CTTTGCAAGC-3′;* CCL2, IL-1β, IL-10 and GAPDH primers were purchased from QIAGEN. For PCR analysis, RNA was transcribed into cDNA using the iScript cDNA synthesis kit (Bio-Rad, Hercules, CA, USA). Quantitative RT-PCR (qRT-PCR) was performed using the StepOne™ Real-Time PCR Detection System (Thermo Fisher) with corresponding primers (Sigma) and Power SYBR Master Mix (Life Technologies). The cycle time values were normalized to GAPDH of the same sample. The expression levels of the mRNAs were then reported as fold changes from sham controls.

### Statistical analysis

One-way ANOVA was used for comparisons between more than two groups. For comparisons between two groups, Student's unpaired t-test was applied. Data from caspase-8-deficient mice were analyzed using two-way ANOVA. Treatment and genetic background, and the interaction between these two were considered the main effects. Statistical analysis was performed using GraphPad Prism6 (GraphPad Inc, La Jolla, CA, USA). All data are presented as mean ± SD with *p* < 0.05 being considered as a significant difference.

## SUPPLEMENTARY MATERIAL



## ABBREVIATIONS

CX3CR1: CX3C chemokine receptor 1
CCR2: C-C chemokine receptor type 2
CCL2: C-C motif ligand 2
DG: Dentate gyrus
GC: Granular cell layer
LysM: Lysozyme M
IR: Irradiation
SGZ: Subgranular zone
