# Supplementary material for: Cranial irradiation induces transient microglia accumulation, followed by long-lasting inflammation and loss of microglia
Source: Oncotarget. 2016 Oct 26;7(50):82305–23. doi: 10.18632/oncotarget.12929 (PMC5347693; doi:10.18632/oncotarget.12929)
Supplement: Supplementary file 1 [file oncotarget-07-82305-s001.pdf]

Cranial irradiation induces transient microglia accumulation, followed by long-lasting inflammation and loss of microglia

Supplementary Material

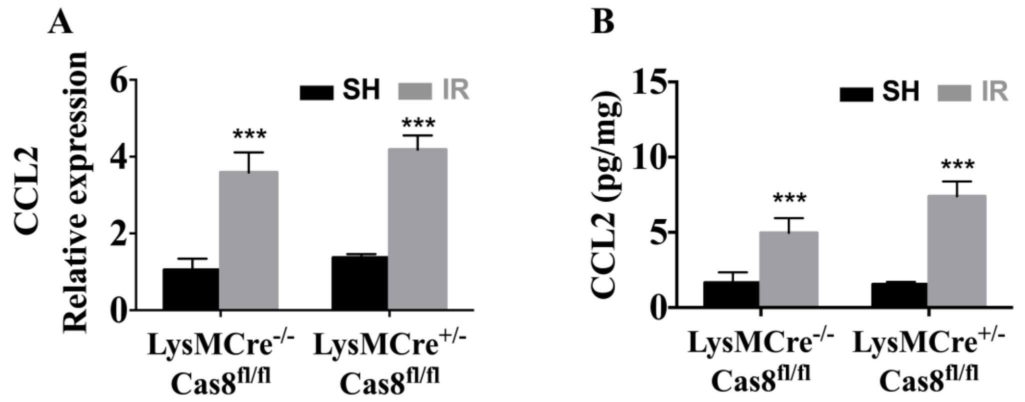

Supplementary figure 1. **Conditional caspase-8 deletion in myeloid cells did not alter irradiation-induced CCL2 expression.** Microglia from sham controls (SH) expressed low CCL2 mRNA levels. Irradiation (IR) increased microglial CCL2 mRNA expression, and this elevation was not impaired in caspase-8deficient mice **(A)**. The CCL2 protein levels in hippocampal homogenates were also elevated after IR, but no difference was found between genotypes. Data are shown as mean ± S.D., n=6 for the sham groups, 7 for the irradiated groups. ***P<0.001 for treatment.
